# Visualizing Dynamic Performance of Lipid Droplets in a Parkinson’s Disease Model via a Smart Photostable Aggregation-Induced Emission Probe

**DOI:** 10.1016/j.isci.2019.10.027

**Published:** 2019-10-18

**Authors:** Lihua Li, Fan Zhou, Qun Gao, Yao Lu, Xingyi Xu, Rong Hu, Zhiming Wang, Mingying Peng, Zhongmin Yang, Ben Zhong Tang

**Affiliations:** 1 State Key Laboratory of Luminescent Materials and Devices, Applied Techniques School of Materials Science and Engineering and Center for Aggregation-Induced Emission, Guangdong Provincial Key Laboratory of Fiber Laser Materials, School of Materials Science and Engineering, School of Physics, South China University of Technology, 381 Wushan Road, Guangzhou 510640, China; 2 Department of Neurosurgery, Beijing Hospital, National Center of Gerontology, Graduate School of Peking Union Medical College, Beijing 100005, China; 3 Department of Orthopedics, Zhujiang Hospital, Southern Medical University, 253 Gongye Road, Guangzhou 510282, China; 4 Department of Chemistry and Hong Kong Branch of Chinese National Engineering Research Center for Tissue Restoration and Reconstruction, The Hong Kong University of Science & Technology, Clear Water Bay, Kowloon, Hong Kong, China

**Keywords:** Disease, Chemical Synthesis, Materials Design

## Abstract

Parkinson’s disease (PD) is a complex neurodegenerative disease affected by diverse factors, and lipid droplets (LDs) are increasingly recognized as major players in PD because of their relevance to neuron activity. However, long-term dynamic changes of LDs and their relative activity remain unclear. Here, an aggregation-induced emission (AIE) probe named 2-DPAN was prepared and employed to visualize dynamic processes of LDs in a 6-hydroxydopamine model of PD for the first time, and LDs' accumulation-peak/plateau-decrease were confirmed. We further found a close relationship between LDs and variation in mitochondrial activity. Strikingly, the progression of cell death was accelerated by lipase, whereas pre-stimulation of LDs by unsaturated fatty acid-oleic acid decreased the death process by inhibiting excessive reactive oxygen species (ROS) and fatty acid production, thereby protecting mitochondria. The utilization of 2-DPAN demonstrates the importance of LDs in neuronal homeostasis, and effective tuning of LDs may prevent or inhibit PD progression.

## Highlights

• 2-DPAN monitors the dynamic changes of Lipid droplets (LDs) in Parkinson disease
• LDs' dynamic change process including three phases, accumulation-plateau-decrease
• LDs' change trend was highly correlated with mitochondrial disruption
• Efficient tuning of LDs could slow the PD progress

## Graphical Abstract

**Figure:**
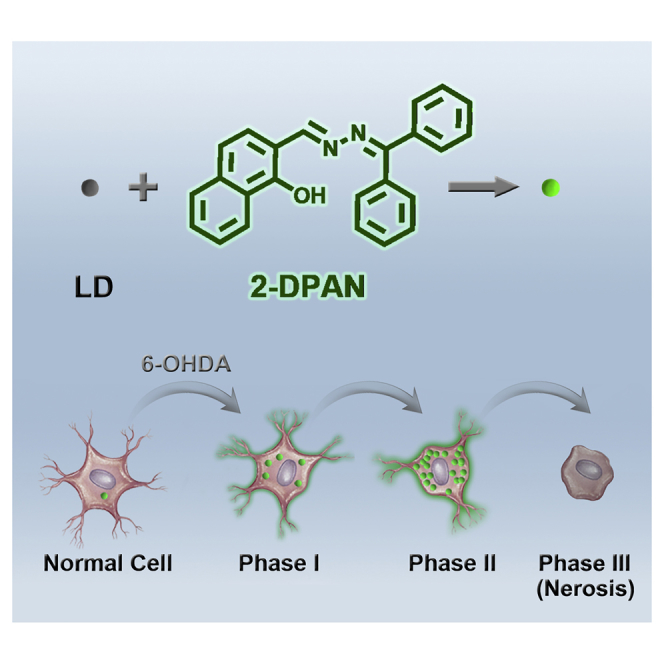

## Introduction

Parkinson’s disease (PD) is one of the most common progressive neurodegenerative disorders that usually occurs in people aged 60 years and above. Characteristics of PD include shaking palsy and involuntary tremble because of the loss of neurons in the substantia nigra pars compacta (SNpc) and the presence of Lewy bodies (Damier et al., 1999, Dauer and Przedborski, 2003). The lifetime risk of developing PD is 1.5%–2% and the average survival time is 15 years, which creates a tremendous burden for the patient, the family, and society. Regrettably, the exact etiology of PD is not well understood (Andreas et al., 2006, Collier et al., 2011, Lees et al., 2009). The main susceptibility factor is age, and widely investigated theories of PD neuropathology include mitochondrial dysfunction, oxidative stress, and dysregulation of lipid metabolism (Gu et al., 2005, Hansruedi, 2009, Shults, 2004).

Lipid droplets (LDs), traditionally recognized as reservoirs of triacylglycerol and cholesteryl ester, have recently been rediscovered as dynamic organelles involved in lipid metabolism, protein storage, signal regulation, and apoptosis (Sally and Parton, 2006). In addition, LDs might act as central players in PD progression. The homeostasis of LDs is highly related to mitochondrial activity and cell states. Mitochondrial dysfunction and oxidative stress can cause LDs accumulation (Liu et al., 2015). In turn, oxidized lipid metabolites promote further mitochondrial dysfunction leading to neuron death and development of PD. Thus, visualizing LDs in realtime would be of great value in the study of PD. However, there is a lack of tools and reagents that can trace LDs over extended time periods. Previous studies show that unsaturated fatty acid (UFA) can inhibit PD progression in both animal models and patients (Bousquet et al., 2008, Hernando et al., 2019). However, the specific action of UFA on LDs and subsequent effects on mitochondria are not known. Thus, the real-time monitoring of LD behaviors in PD remains a challenge.

Agents used to monitor LDs include organic fluorophores, such as the commercial agents Nile Red and BODIPY (Ohsaki et al., 2010, Spandl et al., 2009). Although popular, they have certain disadvantages, such as low photostability, small Stokes shifts, photobleaching, and aggregation-caused quenching (ACQ), which limit their use for long-term tracing in live cells and to monitor disease progression (Daniel et al., 2014, Loudet and Burgess, 2007). Aggregation-induced emission (AIE), a photophysical phenomenon opposite to ACQ, is of interest because of promising practical applications. Usually, fluorogens with AIE characteristics (AIEgens) have a propeller-shaped configuration, such as siloles and tetraphenylethylene (TPE), and are non-emissive when molecularly dissolved because of their activity of intramolecular vibration and rotation from peripheral free phenyl group, which is the main way to dissipate the energy of excited states in non-radiative transition process. When they aggregate to nanoparticles or solid films, these motions would be restricted originating from packing effect, and their emission intensified remarkable upon because the radiation transition process becomes the dominant mode of energy release. This mechanism for AIE phenomena is vividly described as the restriction of intramolecular motion (RIM); sometimes they are classified as the restriction of intramolecular rotation and the restriction of intramolecular vibration, which have been proved by systematical experimental investigation and theoretical simulation (Mei et al., 2015). In addition, a large number of luminescent components form nanoparticles, and the inner probe molecules can be protected from photobleaching and photooxidation, resulting in high photostability of the probes. Therefore, it is envisioned that employing AIEgens to probe dynamic changes of LDs in PD procession could be a promising approach with high sensitivity and stability.

**Scheme 1:**
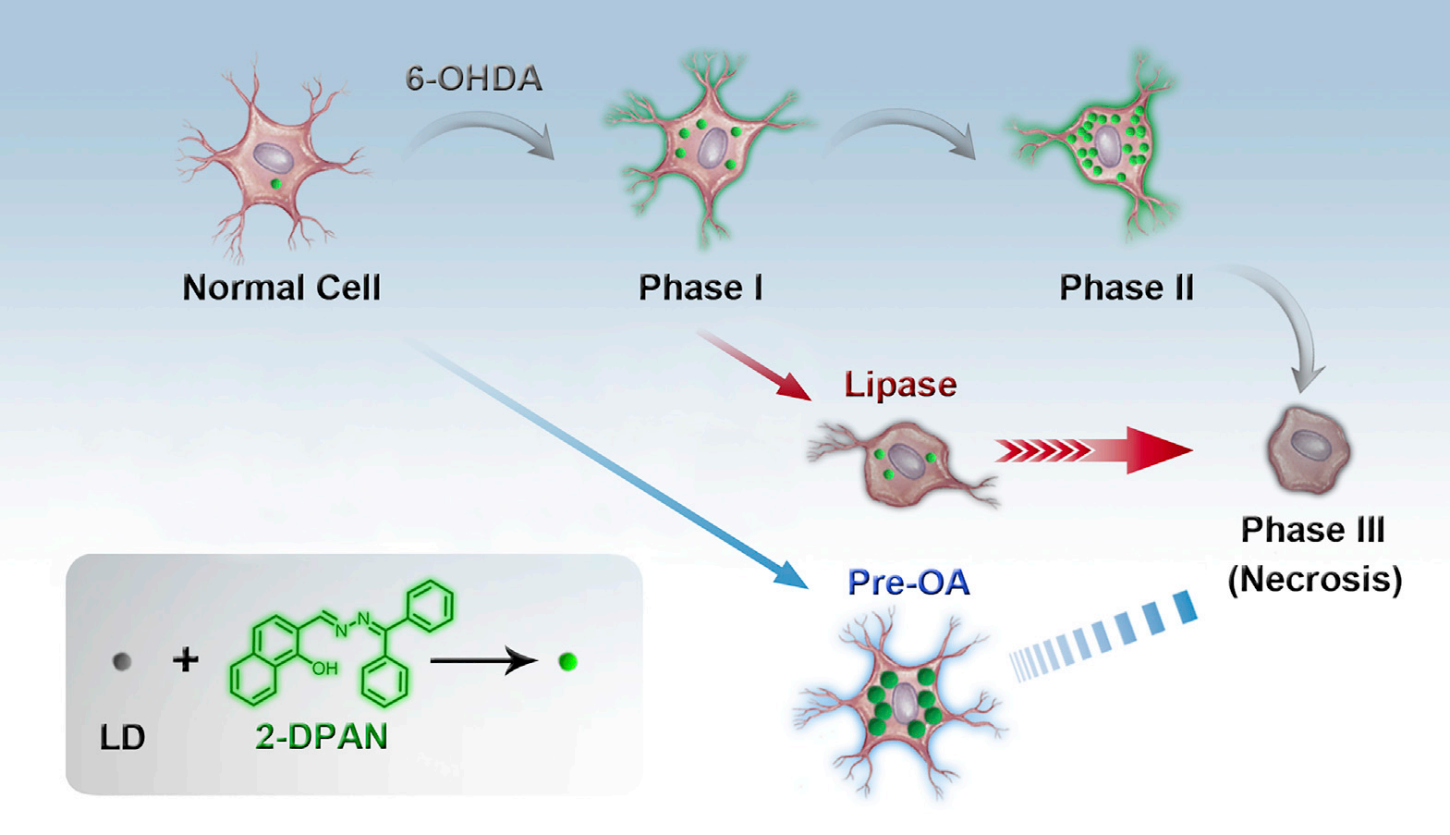
Real-Time Monitoring LDs' Behaviors in 6-OHDA-Induced PD Model Using 2-DPAN as a LDs-Specific Probe

According to our previous report on lipid droplets-targeting 2-(((9H-fluoren-9-ylidene)hydrazono)-methyl)phenol (FAS) (2-(((diphenylmethylene)hydrazono)methyl) -phenol) (DPAS), we found that keto-salicylaldehyde hydrazine (KSA) derivatives usually exhibit typical AIE-activity with excited-state intramolecular proton transfer characteristics (Zhang et al., 2019). Unlike the common AIEgens based on RIM process via packing effect as described earlier, KSA usually forms additional six-membered ring structures in the aggregated state with the aid of intramolecular hydrogen bonding, which activates the RIM and then gives rise to fluorescence enhancement. What's more, these KSA-based probe molecules with some hydrophilic ability enter cells more easily than simple aryl derivatives (such as TPE), and they could effectively recognize specific organelles through introducing some appropriate targeting modification (Hu et al., 2018, Wang et al., 2016, Wang et al., 2018a, Wang et al., 2018b, Zhao et al., 2017). In this study, we prepared a DPAN-based probe, 2-DPAN, by inserting a 2-naphthalene group instead of phenyl group for enhancing luminous efficiency, which could selectively target and accumulate in cellular LDs and emit bright green fluorescence. Combining high photostability and excellent biocompatibility, it has potential for studying dynamic LDs changes in a 6-OHDA-induced model of PD. Importantly, we report real-time dynamics of LDs after stimulation: LDs rapidly accumulated in the first stage, then reached a plateau in the second stage, and they were released from damaged cells, lastly. At the same time, the correlation between LDs and mitochondria was investigated at selected intervals and showed a negative relationship between LDs and mitochondrial activity in the first II phases. In addition, lipase was used to study the effect of inhibiting LD accumulation on cell activity, and it caused even worse damage to cells. When cells were pre-treated with oleic acid (OA), the newly generated LDs slowed the cell death progress. We further showed that inhibition of PD progression was achieved by protecting mitochondria through the inhibition of reactive oxygen species (ROS) production and excessive fatty acid synthesis (Scheme 1). Our results indicated that controlling the equilibrium of LDs can have a positive effect on the protection of cells in 6-OHDA-induced PD and that 2-DPAN is a promising tool to dissect the relationship between LDs and PD progression.

## Results

### Synthesis and Photophysical Properties of 2-DPAN

**Figure 1:**
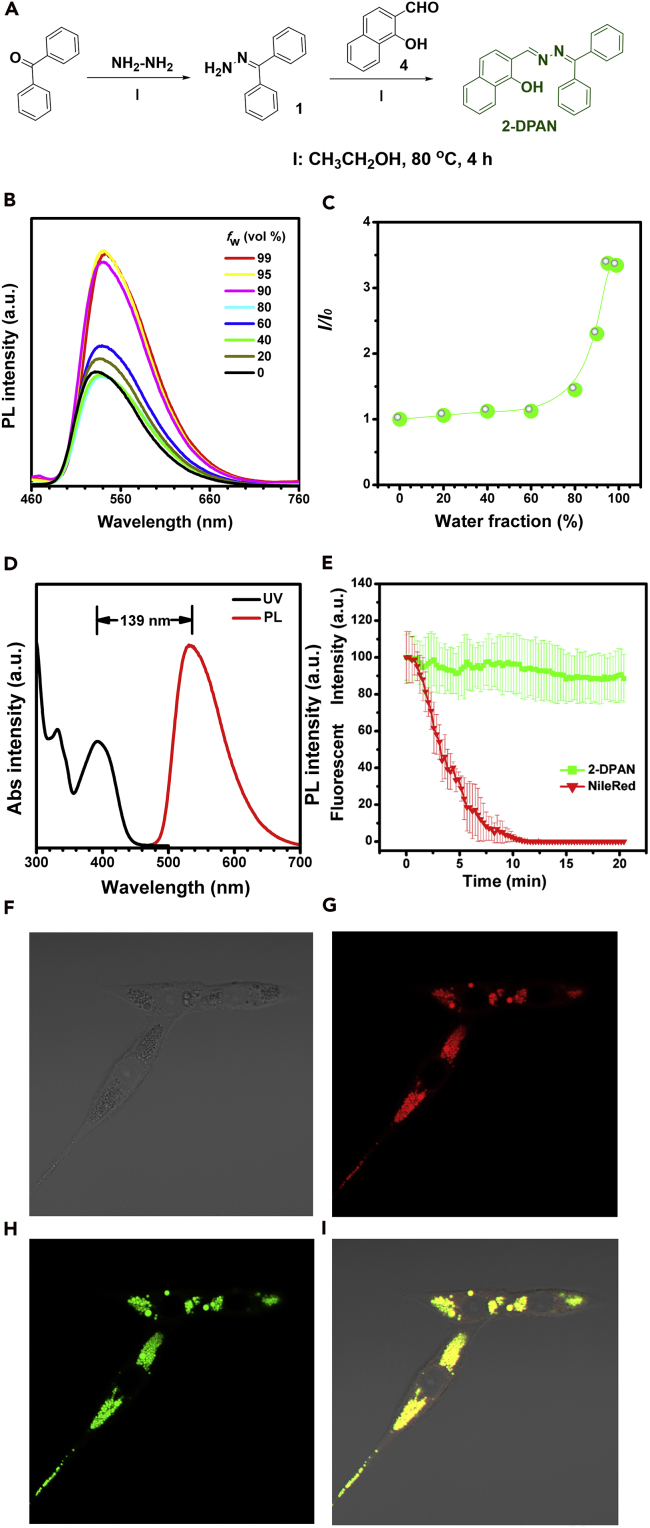
Characterization of 2-DPAN (A) Synthetic route of 2-DPAN. See also Figure S1. (B) Photoluminescence spectra of 2-DAPN in tetrahydrofuran (THF)/water mixtures with different water ratios. ([2-DPAN] = 10 μM. Excitation wavelength at 405 nm). (C) Plots of *I*/*I*_*0*_ versus water fractions of 2-DPAN, where *I*_*0*_ is the emission intensity in THF. (D) The absorption spectra and photoluminescence spectra of 2-DAPN with Stokes shift of 139 nm. (E) Photostability of 2-DPAN and Nile Red (commercial LDs tracker) under exposure to 405 and 565 nm laser with increasing number of scans (power intensity of 99%), respectively. Cells co-stained with 2-DPAN and Nile Red. (F) Bright field. (G) Red fluorescence collecting channel for Nile Red. (H) Green fluorescence collecting channel for 2-DPAN. (I) Merge imaging, the SH-SY5Y cells were pre-induced by OA for 6 h, where the concentration of OA was 1 mM.)

With reference to our previous reports on the structure-properties relationship of KSA-based derivatives and the performance requirements of probes for PD research, we designed and prepared 2-DPAN as an LD-specific probe, where the introduction of a 2-naphthalene group effectively increased the luminescence efficiency in the aggregate state (Zhang et al., 2019). The detailed procedure for its synthesis and characterization are given in Figures 1A and S1. Aggregates gradually formed by the addition of a poor solvent (water) to a good solvent (tetrahydrofuran [THF]), giving rise to significantly improved photoluminescence (PL) intensity (the plot of *I*/*I*_0_), indicative of AIE-activity (Figures 1B and 1C). Moreover, we have investigated the stability of 2-DPAN at different organic solvents and pH solutions. As shown in Figures S1B and S1C, there were no significant changes in PL spectra and *I*/*I*_0_, illustrating high stability of the molecular structure in 2-DPAN. At fraction of water (*f*_w_) = 99%, its fluorescent intensity reached a peak with a quantum yield of 7.1%, indicating that excellent fluorescence performance was maintained in an aqueous environment. Similar to most AIEgens based on the excited-state intramolecular proton transfer process, the 2-DPAN peak was at approximately 550 nm without significant shift when the external environment changes (*f*_w_). Such a stable emission peak is very beneficial for long-term monitoring compared with donor acceptor (D-A) fluorescent probes (some blue/red-shift is usually observed when the polar environment changes, e.g., for Nile-red). As a typical keto-emission, a large Stokes shift (139 nm) between absorption and emission peaks (Figure 1D) was also observed, and self-absorption was efficiently avoided in the bioimaging field (Wang et al., 2018a, Wang et al., 2018b).

### Photostability, Specificity, and Biocompatibility of 2-DPAN

To develop 2-DPAN for tracking PD progression, first its specificity, photostability, and biocompatibility were investigated. For investigating LD-specific probes, OA is suitable for inducing cells to produce more LDs for co-staining experiments. Here, SH-SY5Y cells were employed and pre-co-cultured with OA (1 mM) for 6 h to make LDs more visible. Subsequently, these cells were co-stained with the commercial product (Nile Red) and 2-DPAN. Under a confocal microscope for 20 min with 60 scans (laser density of 99%), the fluorescent intensity of 2-DPAN-dyed cells did not diminish, whereas more than 95% of signal was lost from the Nile Red group, indicating excellent photostability of 2-DPAN (Figure 1E). As shown in Figure 1F, the LDs in cells were clearly visible under confocal microscopy following excitation at wavelengths of 405 nm (for 2-DPAN) and 532 nm (for Nile Red), and Pearson colocalization efficiency was 96%, illustrating a high specificity of 2-DPAN for labeling LDs (Figures 1G–1I). In addition to this advantage of specificity in labeling of LDs, there was little leakage phenomenon in 2-DPAN than Nile Red, which would usually penetrate other organelles with time. The stability and specificity of 2-DPAN is, therefore, superior to the commercial dye. Moreover, 2-DPAN also exhibited good biocompatibility even with a very high working concentration (5 mM) (Figure S2A). Thus, the excellent performance of 2-DPAN makes it a promising probe for extended tracing of the PD process.

### Dynamic Monitoring of LDs in a PD Model

The involvement of LDs in disease is increasingly appreciated (Liu et al., 2015), which stimulated our interest in investigating dynamic changes of LDs in PD. We first examined the long-term photostability and toxicity of 2-DPAN by real-time monitoring of LDs in SH-SY5Y cells by co-culture under confocal microscopy. As shown in Figure S3, there were no significant changes in LD fluorescence or LD accumulation during the observation period. In addition, the cells maintained normal shapes and steady fluorescence with time, demonstrating that 2-DPAN can be visualized and monitored for extended periods.

**Figure 2:**
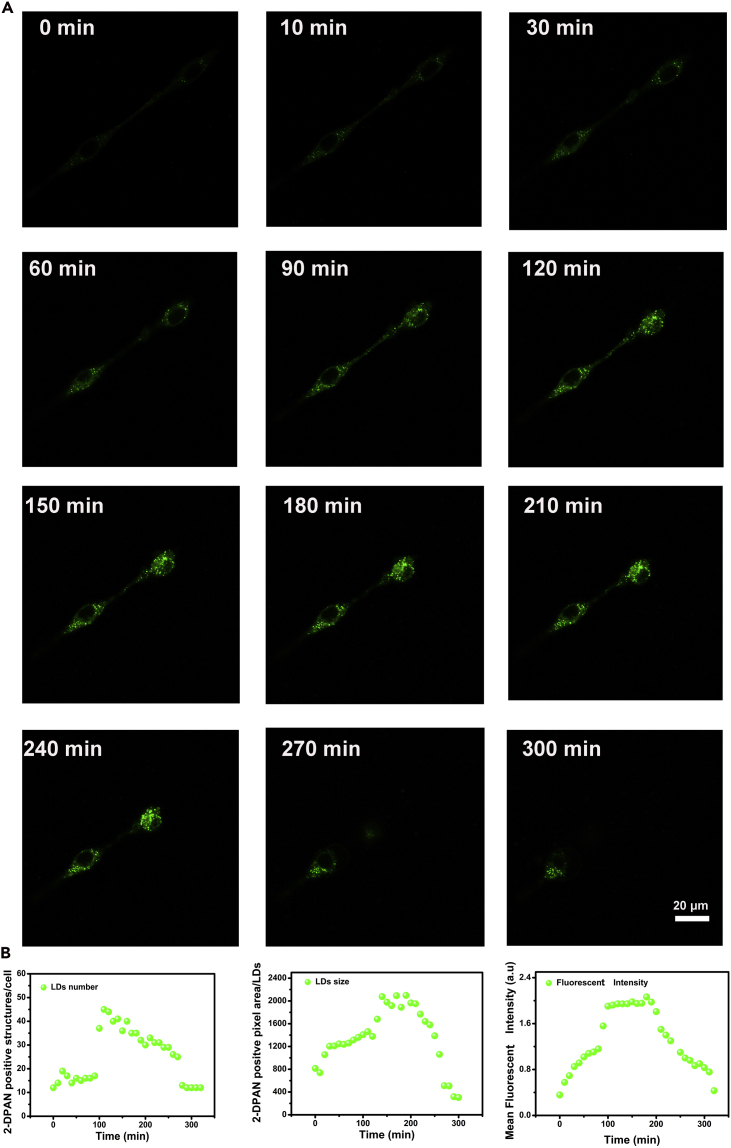
Dynamic Changes of Lipid Droplets in 6-OHDA Induced PD Model (A) Fluorescence images of LDs in 6-OHDA induced cells at varied times. See also Figures S2 and S3. (B) LDs number, LDs size, and mean fluorescent intensity of LDs (analyzed by Zen software in Confocal Microscopy). (Excitation wavelength at 405 nm, and the growth curve from 0 to 320 min with step of 10 min; the represented images were selected at every 30 min in Figure 2A).

Second, we established a 6-hydroxydopamine (6-OHDA)-induced PD model in SH-SY5Y cells (model details can be seen in Figures S2B and S2C). In control, non-PD model cells, few LDs were observed and the number did not change over time (Figure S3). However, in 6-OHDA-induced PD model cells, the number of LDs and their size increased over the initial 100 min after 6-OHDA treatment (Figures 2A and 2B). Furthermore, the fluorescent intensity from LDs (stained by 2-DPAN) increased logarithmically (Figure 2B), indicating rapid accumulation of LDs, which was consistent with reported LD formation in the early stage of apoptosis (Boren and Brindle, 2012). The shape of LDs and cellular pseudopods also varied at this initial stage. In the next period from 100 to 210 min, cells suffered from severe deformation as LD numbers reached their peak, indicating that LDs accumulation occurs before cell changes and that LDs may be a potential target for halting PD progression.^12,15^ After co-culture for 300 min, cells mostly lost their shape and the number of LDs decreased substantially. After 24 h of incubation with 2-DPAN and 6-OHDA, cells were seriously damaged with few LDs; however, these remaining LDs displayed strong fluorescent intensity, illustrating the ultralong labeling ability of 2-DPAN (Figure S4) and the close relationship between LDs and cell death. We further assessed the effects of 2-DPAN on 6-OHDA-induced cells to eliminate any adverse effects; there were negligible effects on the cell model, illustrating the excellent labeling characters of 2-DPAN (Figure S2D). The changes in LDs numbers, which took the pattern accumulation-plateau-release, were associated with cell viability, further indicating the high correlation between LDs and PD progress.

### 6-OHDA-Induced Changes between Mitochondria and LDs

**Figure 3:**
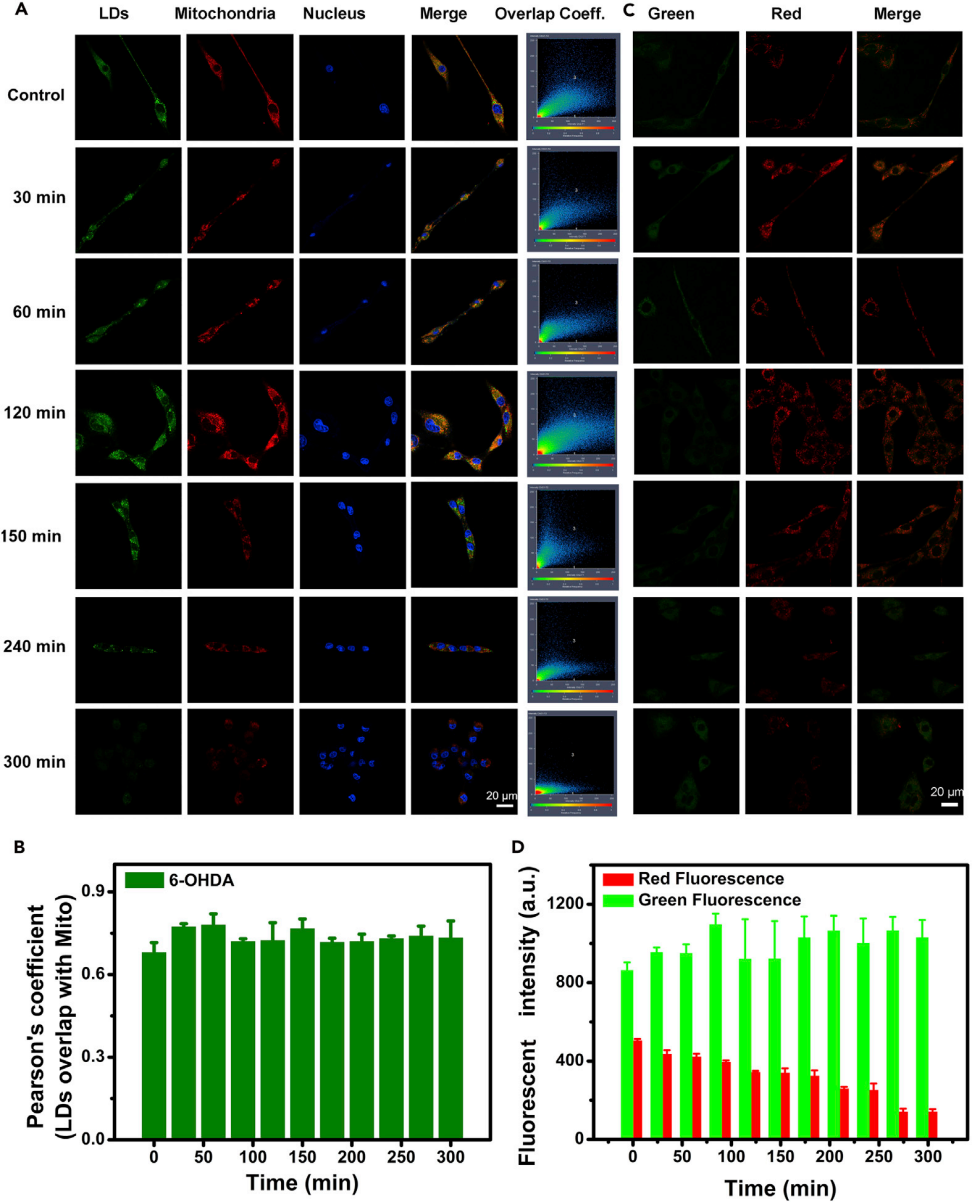
LDs and Mitochondrial Changes in 6-OHDA-Induced Cells (A) Confocal fluorescence images of 6-OHDA-induced PD cells at varied time. (Co-stained with 2-DPAN for Green, Mito Tracker for Red, and Hochest 33342 for Blue). See also Figure S4. (B) Quantification of the correlation between green signal (LDs) and red signal (mitochondria) during 6-OHDA-induced PD process. n = 4, data are represented as mean ± SEM. (C) Confocal fluorescence images of JC-10 labeled cells in 6-OHDA-induced PD cells at varied times (Ex/Em: Green, 488 nm/525–540 nm; Red, 543 nm/590–700 nm). (D) Fluorescent intensity changes monitored by a fluorescence microplate reader (Green, Ex/Em = 490/525 nm; Red, Ex/Em = 540/590 nm.). n = 4, data are represented as mean ± SEM.

Mitochondrial damage is widely regarded as a key factor in PD progression and is notably advanced by accumulation of lipid and cholesterol oxidation products (Kinghorn and Castillo-Quan, 2016). Mitochondrial dysfunction then results in increased synthesis of lipid from excess fatty acid, generating more LDs (Lin and Beal, 2006). Here, we monitored the correlation between LDs and mitochondria at selected intervals and showed the negative relationship between them and the mitochondrial activity (mitochondrial cell membrane and shapes). To visually address the dynamic relationship between LDs and mitochondria, LDs, mitochondria, and nuclei were co-stained using 2-DPAN, Mito Tracker, and Hoechst 33342, respectively. Imaging data were collected every 30 min during 300 min in 6-OHDA PD model cells. Regrettably, the observed region could not be fixed to a certain area because of low photostability (photobleaching effect) and toxicity of the commercial dyes (Mito Tracker and Hoechst 33342). Therefore, an alternative strategy was attempted. The probe with stable optical properties (2-DPAN) was used in continuous *in situ* observation mode, and the other optical probes were only applied before the test. We monitored corresponding changes in mitochondria, as shown in Figure 3A. LDs fluorescent intensity increased with mitochondrial dysfunction and cell damage, and then LDs fluorescent intensity decreased with increasing mitochondrial defects and cell death. More importantly, we found that all LDs were in close proximity to mitochondria during the 6-OHDA-induced process; the overlap of LDs and mitochondria was above 60% (Figure 3B), illustrating potential transfer of energy and fatty acids between them, and similar findings have been described by Rambold et al. (2015). The viability of cells was highly correlated with mitochondrial activity and LDs status. We also monitored mitochondrial membrane potential. As shown in Figures 3C and 3D, the fluorescence emission shifted from red to green with time, indicating transfer from JC-10 dimer to JC-10 monomer. These results indicated the loss of mitochondrial membrane potential during the 6-OHDA-induced process.

**Figure 4:**
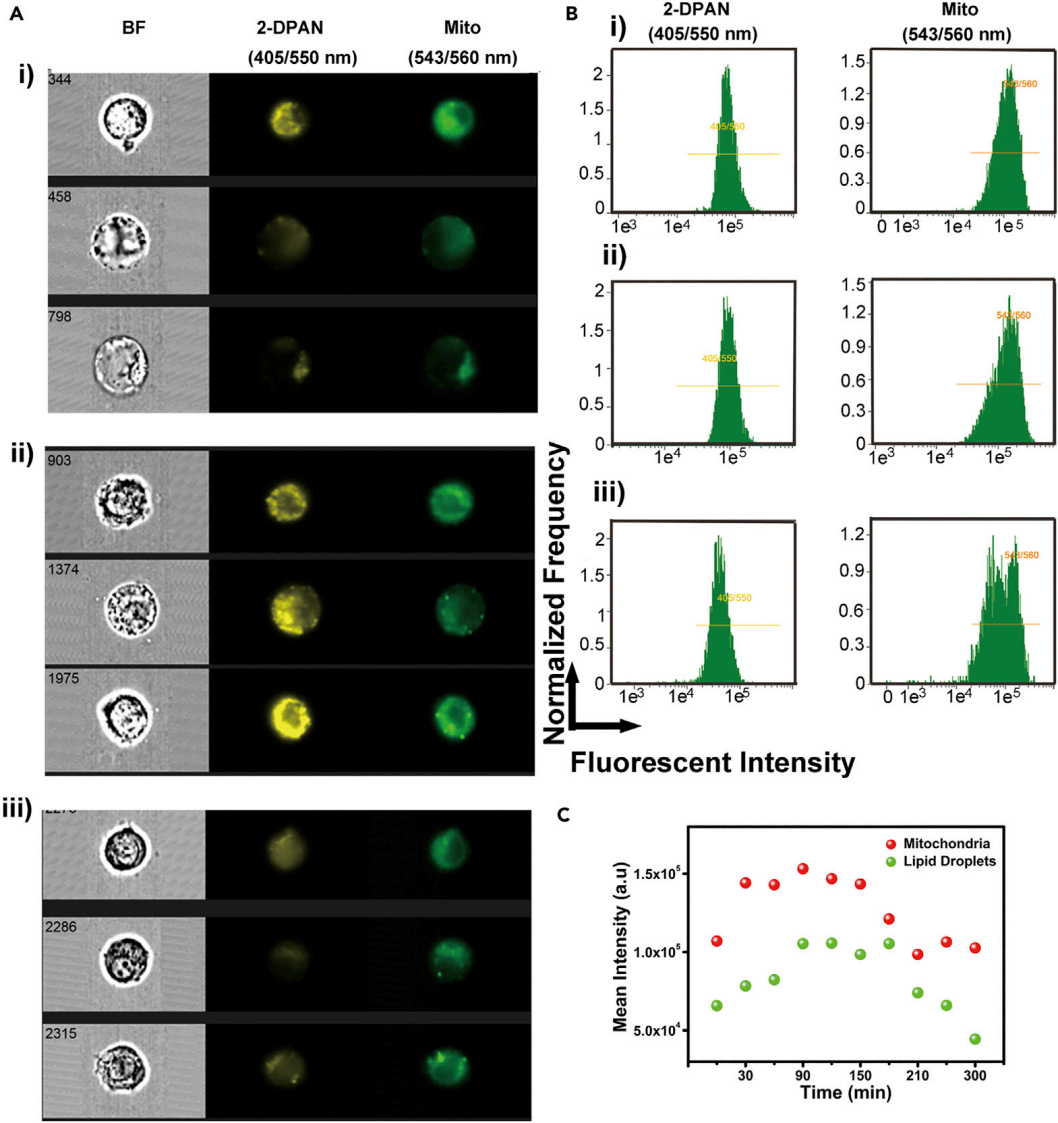
The Fluorescent Intensity Changes of Mitochondria and LDs by Imaging Flow Cytometry (A) Representative fluorescence images of mitochondria and LDs at (i) 30 min, (ii) 150 min, and (iii) 300 min. (B) Fluorescence diagram of flow cytometry at (i) 30 min, (ii) 150 min, and (iii) 300 min. (C) Quantitative mean fluorescent intensity of LDs and mitochondria every 30 min during 300 min in 6-OHDA-induced process.

To further investigate the correlation between changes to LDs and mitochondria, we studied the variation of fluorescent intensity by flow cytometry. As shown in Figure 4A, cells were initially intact with visible fluorescence from LDs and mitochondria. After 300 min, cells were shrunken with weak fluorescence. Detailed changes are shown in Figures 4B and 4C. LDs (405 nm excitation, 505–560 nm emission) and mitochondrial fluorescence (561 nm excitation, 560–595 nm emission) in 6-OHDA-treated cells showed a similar trend, increase-plateau-decrease, which was consistent with the above-mentioned results. The LDs accumulation process proceeded when the mitochondrial activity was decreasing, illustrating that excess lipid in LDs may be synthesized from the damaged mitochondria. Then with cell deformation, LDs rapidly lost their fluorescence (Figure 4C). In addition, the relationship between mitochondria and LDs in PD progression was not completely linear but mirrored the cell state.

### Induction or Inhibition of LDs Has Opposite Effects on PD

**Figure 5:**
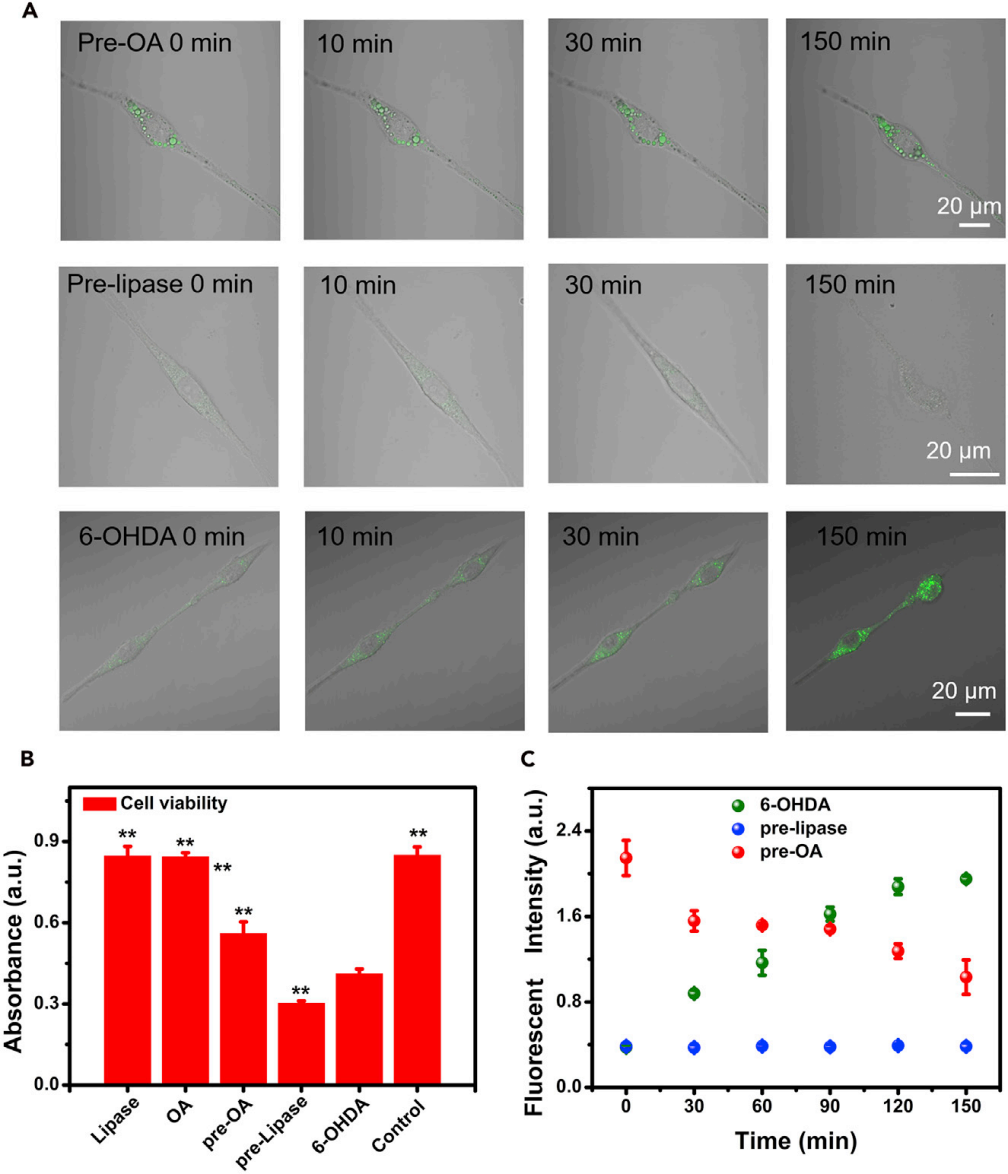
The Effects of Lipase and OA on 6-OHDA-Induced PD Cells (A) Visual images of cells pre-cultured with OA (1 mM) for 6 h, and then treated with 6-OHDA (Pre-OA); pre-cultured with lipase for 6 h, and then treated with 6-OHDA (Pre-lipase); treated only with 6-OHDA (6-OHDA). (B) Cell viability of different groups, data are expressed as means ± standard deviation of triplicate determinations. All groups were compared with the 6-OHDA treated group. n = 4, data are represented as mean ± SEM. *p < 0.05. **p < 0.01. (C) Mean fluorescent intensity of LDs in different groups. n = 4, data are represented as mean ± SEM. See also Figure S5.

The aforementioned results were encouraging. The enhancement or reduction of LD accumulation causes mitochondrial protection or dysfunction, respectively, resulting in inhibition or enhancement of the PD process. Here, OA (to stimulate more LDs) and lipase (to decompose LDs) (Figure S5) were employed to investigate changes in LDs and cell viability in the PD process. Lipase was pre-cultured with cells for 6 h before 6-OHDA was added. It significantly accelerated cell demise, and fewer LDs were present. Cells were seriously damaged and lost their viability (Figure 5A). In contrast, when cells were pre-treated with OA, an obvious increase in the number of LDs and effective inhibition of the 6-OHDA-induced PD process were observed (p < 0.01). After 150 min, OA-treated cells retained their original shape, showing superiority over other groups. The cell viability (Figure 5B) and fluorescent intensity of LDs (Figure 5C) were also investigated, indicating the protective effect of OA and destructive effect of Lipase on the PD model. In addition, we added lipase or OA in the logarithmic phase of LDs accumulation period to inhibit/induce LDs synthesis. Surprisingly, we found that lipase can significantly accelerate cell demise (p < 0.01) with fewer LDs. Cells were seriously damaged and lost their viability soon (Figure S5C). There was negligible effect of OA on PD progress. Overall, the lipase increased fatty acid hydrolysis and consumption, disrupted membrane integrity, dysregulated lipid metabolism, and accelerated cell death. In contrast, OA increased the number of LDs, which provided a protective effect for cells from 6-OHDA. These results hint that augmenting LDs would inhibit PD progression.

### The Potential Protective Mechanism of Pre-OA in the 6-OHDA-Induced Model of PD

**Figure 6:**
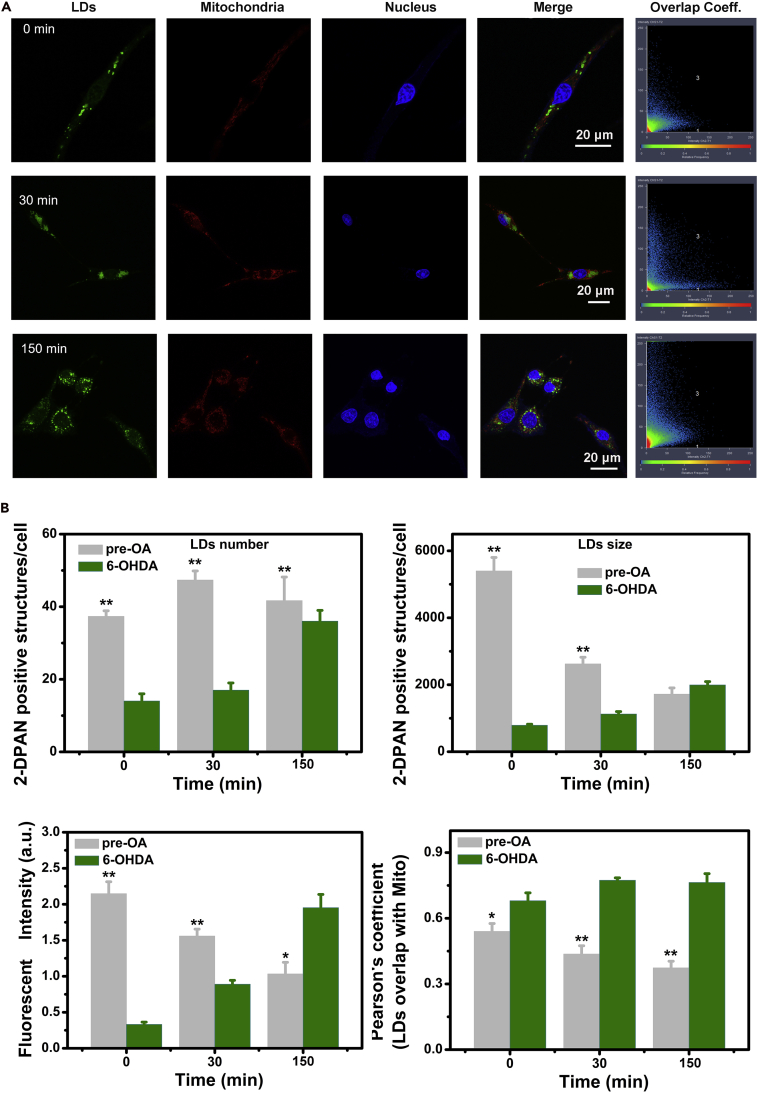
The Potential Relationship between LDs and Mitochondria (A) The distribution of LDs and mitochondria in cells pre-treated with OA for 6 h, then treated with 6-OHDA and captured at 0, 30, 150 min under confocal microscopy. (Green for LDs using 2-DPAN, Red for mitochondria using Mito tracer, and Blue for cell nucleus using Hochest 33342.). (B) LDs numbers, LDs size, mean fluorescent intensity of LDs, and the overlap areas (Pearson coefficient) between LDs and Mitochondria at 0, 30, 150 min of pre-OA treated group and control (untreated) group. Data are expressed as means ± standard deviation of triplicate determinations. n = 4, data are represented as mean ± SEM. *p < 0.05; **p < 0.01; n.s., not significant. See also Figure S6.

We then tested whether pre-stimulation of LDs was specific for protecting mitochondria or reducing oxidative stress in 6-OHDA-induced injury. To address this issue, differences in LDs and mitochondria distribution between pre-OA-treated and untreated groups were investigated. A significantly increase in LDs number, size, and fluorescent intensity (p < 0.01) occurred in cells pre-incubated with OA (Figures 6A and 6B). The overlap of LDs and mitochondria in pre-OA treated and control groups were then investigated. OA was first stored in LDs in close proximity to mitochondria. When 6-OHDA was introduced, LDs size and fluorescent intensity declined in the pre-OA group. Simultaneously, the Pearson coefficient efficiency of LDs and mitochondria and ROS production were significantly lower in the pre-OA group compared with the 6-++OHDA-only treated group (p < 0.01), illustrating a prominent inhibition of fatty acid (FA). Furthermore, a significantly decrease of ROS (Figure S6) was also found in the pre-OA treated group, illustrating that a combined protection effect was achieved by inhibiting both FA and ROS synthesis. In contrast, high levels of LDs and ROS were formed in the untreated group. Furthermore, the LDs were in close proximity to the mitochondria in the 6-OHDA-treated group, with a high mitochondria and LDs overlap value (>0.7), indicating sustained production of LDs and ROS (Figure S6). The oxidative stress further aggravated mitochondrial dysfunction and accelerated cell injury in the 6-OHDA-treated group. We also employed antioxidants (astaxanthin, Asx) to clear ROS and prove the excessive ROS hypothesis. Asx could significantly improve the cell viability (Figure S5D). Thus, pre-OA may protect cells from 6-OHDA-induced death by reducing ROS and LDs production to protect mitochondria. Similar results have been observed in animal experiments with UFA providing a protective effect against PD; however, there was a lack of tools to explore the relationship among UFA, LDs, and mitochondria (Carole et al., 2010, Hernando et al., 2019). We have shown that the photostable AIEgen, 2-DPAN, can be used to address these issues, and our results indicate the possible mechanism of UFA protection for PD.

## Discussion

An ultra-stable AIEgen probe, 2-DPAN, was synthesized for monitoring the behavior of LDs in 6-OHDA-induced PD model cells. Luckily, the LDs' dynamic change process with three phases of accumulation-plateau-decrease was first reported, and this trend was highly correlated with mitochondrial disruption by detail imaging experiment. After introducing lipase and OA to inhibit or stimulate LDs, respectively, we surprisingly found that lipase could seriously injure cells and accelerate the PD process. In contrast, OA pre-treatment significantly prevented cell death by inhibiting excessive ROS and augmenting LD formation to protect mitochondria. These results indicate that the real-time behavior monitoring of LDs was very important and necessary in the 6-OHDA-induced PD early prevention process, and the AIEgens represented by 2-DPAN may be an useful tool for studying LD-related diseases owing to their excellent specificity, photostability, and biocompatibility.

### Limitations of the Study

The AIE probe 2-DPAN presented here is able to label LDs *in vitro* vividly with high stability and for ultralong time, and we found the dynamic changes of LDs in 6-OHDA-induced PD progress. In addition, we also found the close relationship between mitochondria and LDs in PD cells. The probe further verified the importance of LDs homeostasis in PD progress. We believe our study will be a great appeal to researchers in probe designing, cell biology, and neuroscience. Owing to the complex structure and depth of brain, the LDs changes in SNpc and SN area have not been monitored. Future work should be carried out on living animals. In addition, as saturated fatty acids and UFAs have different effects on human's PD progress, the uptake of UFA may exert its effect on LDs maintenance in living PD animals.

## Methods

All methods can be found in the accompanying Transparent Methods supplemental file.

## Supplemental Information

Document S1. Transparent Methods and Figures S1–S6
