# Supplementary material for: Visualizing Dynamic Performance of Lipid Droplets in a Parkinson’s Disease Model via a Smart Photostable Aggregation-Induced Emission Probe
Source: iScience. 2019 Oct 18;21:261–72. doi: 10.1016/j.isci.2019.10.027 (PMC6838505; doi:10.1016/j.isci.2019.10.027)
Supplement: Document S1. Transparent Methods and Figures S1–S6 [file mmc1.pdf]

ISCI, Volume *21*

Supplemental Information

Visualizing Dynamic Performance of Lipid Droplets in a Parkinson's Disease Model via a Smart Photostable Aggregation-Induced Emission Probe

Lihua Li, Fan Zhou, Qun Gao, Yao Lu, Xingyi Xu, Rong Hu, Zhiming Wang, Mingying Peng, Zhongmin Yang, and Ben Zhong Tang

Supplemental Information

Supplemental Figures

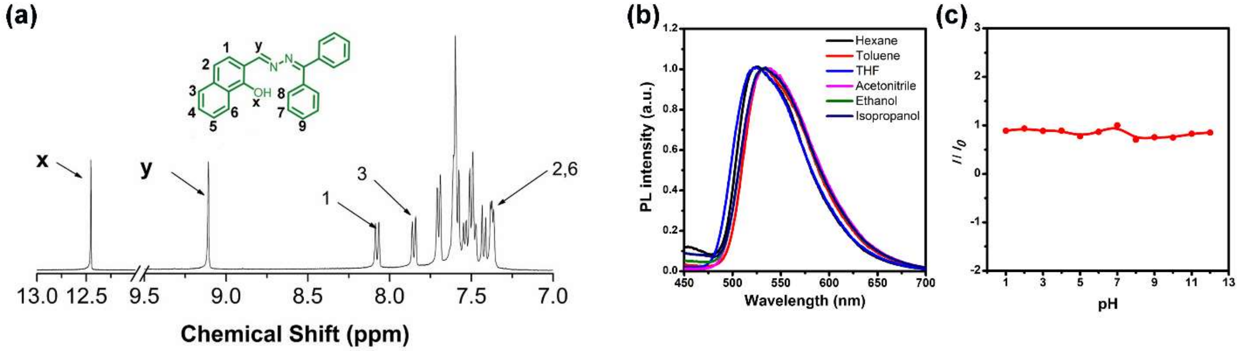

Figure S1. The characterization of 2-DPAN, Related to Figure 1.

(a)^{1}H-NMR spectra of the 2-DPAN. (b)The photoluminescence spectra of 2-DAPN in different organic solvents. (c) Plots of I/I_0 vs water fractions of 2-DPAN at different pH value, where I_0 is the emission intensity in THF.

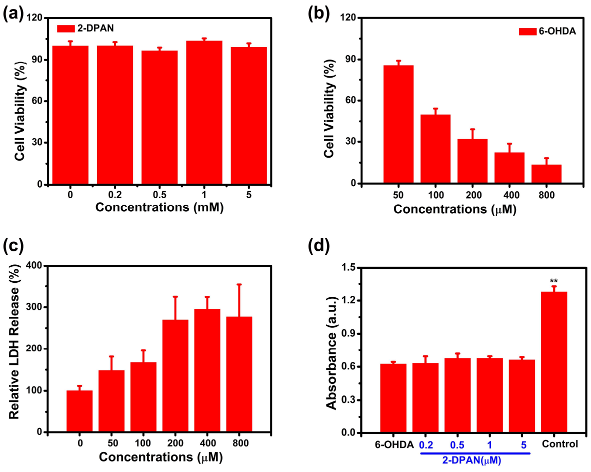

Figure S2. Cytotoxicity of 2-DPAN and 6-OHDA on SH-SY5Y cells after co-cultured for 24 h, Related to Figure 2.

Cell viability of the 2-DPAN (a), Cell viability (b) and LDH release rate (c) in the supernatant of SH-SY5Y cells with varied 6-OHDA concentrations. (d) The effect of 2-DPAN on 6-OHDA induced PD cells.

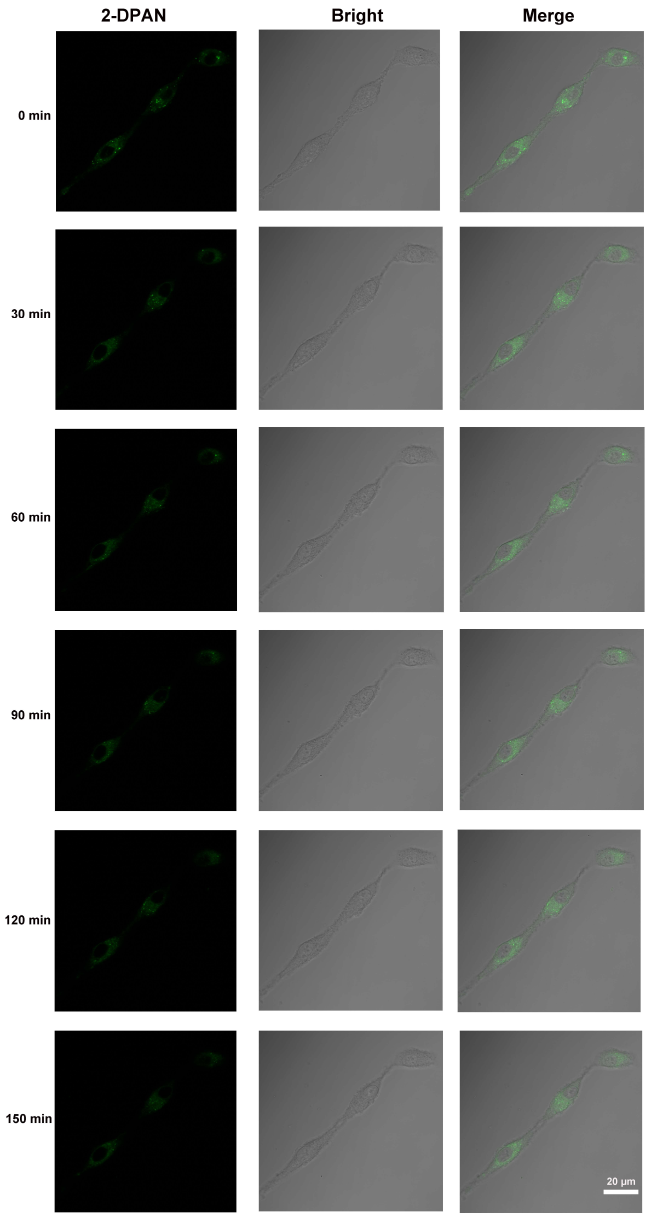

Figure S3 Images of LDs in blank cells at varied times (Excitation wavelength at 405 nm, Scale bar = 20 μm, and the growth curve from 0-320 min with step of 10 min, the represented images were selected), Related to Figure 2.

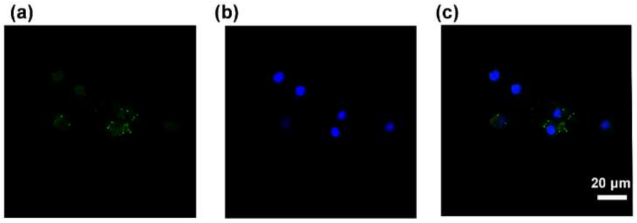

Figure S4. Confocal images of 6-OHDA induced cells for 24 h, related to Figure 3.

(a) 2-DPAN was co-cultured with cells for 24 h. (b) Cell nuclear stained with Hochest 33342 for 10 min in advance. (c) The merged images of (a) and (b).

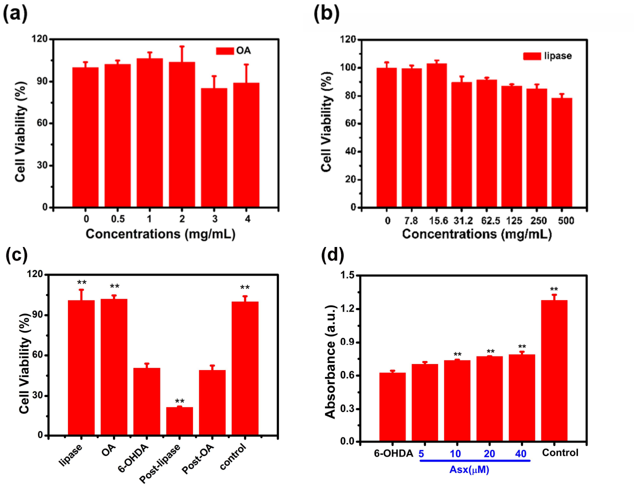

Figure S5. Cell viability of different treatment groups, related to Figure 5.

Cell viability of (a) Oleic acid with different concentrations. (b) Lipase with different concentrations, the lipase activity is 300,000 U/g.(c) Cell viability of different groups. Lipase and OA were added in the LDs accumulation stage in PD progress (at 30 min after 6-OHDA added). (d) The cell protection effect of antioxidation (astaxanthin, Asx) on 6-OHDA induced PD model**.**

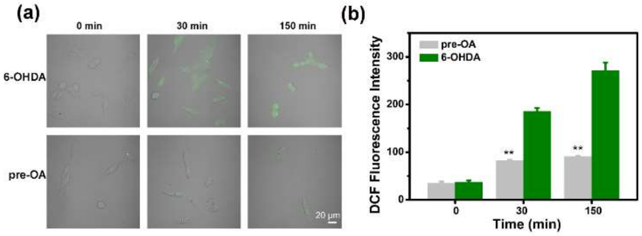

Figure S6. Quantitative of ROS in control and pre-treated OA in 6-OHDA induced PD model, related to Figure 6.

(a) Confocal images of cells co-cultured with DCFH-DA at different time intervals. (b) Mean DCF fluorescence in pre-OA and 6-OHDA treated groups in selected time intervals. Data are expressed as means ± SEM. *$p < 0.05$; ** $p < 0.01$.

Transparent Methods

Synthesis

The synthesis of compound 1 was carried out as previously reported [11e]. Benzophenone from cheap commercial product was refluxed with excess hydrazine hydrate in ethanol for 4 h to afford white needle crystals of M1 in 95% yield after cooling to room temperature. The Compounds M1 (7.85 g, 40 mmol) and 1-hydroxy-2-naphthaldehyde (7.23g, 42 mmol) were dissolved with THF, ethanol and 2 drops of glacial acetic acid. The reaction was carried out for 4 hours under reflux. The residual solvent was removed by vacuum distillation and then purified by silica gel chromatography with DCM: PE (1:6) as eluent to afford 1-DPAN with nearly 90% yields.

^{1}H NMR ($CDCl_3$, 500 MHz), δ(TMS, ppm): 12.44 (s, 1H), 8.93 (s, 1H), 8.27-8.25 (d, J = 10.0 Hz, 1H), 7.79-7.77 (d, J = 10.0 Hz, 2H), 7.73-7.71 (d, J = 10.0 Hz, 1H), 7.56-7.50 (m, 4H), 7.47-7.36 (m, 6H), 7.33-7.29 (m, 2H).

Photostability test

Cells co-cultured with 2-DPAN and Nile Red for 20 min, then they were imaged by confocal microscope (Zeiss laser scanning confocal microscope LSM7 DUO) with conditions: for 2M-DPAN, excitation wavelength = 405 nm and emission filter = 468-560 nm; for Nile Red, excitation wavelength = 561 nm and emission filter = 573-696 nm. For co-staining, the SH-SY5Y cells were pre-cultured with oleic acid (1 mM) for 6 h to induce more LDs, then co-stained with Nile Red and 2-DPAN (2 μM).

Real-time monitoring LDs in cells

Cells were seeded in a 35 mm Petri dish with a coverslip at 37 ºC overnight, then 6-OHDA (0, 100 μM) and 2-DPAN (5 μM) were added to the medium, respectively. The Petri dish were placed into the confocal microscopy (37ºC, 5% CO_2) with a continuous observation for 300 min. The excitation wavelength was 405 nm and the scanning interval was every 10 min. LDs fluorescence intensity, overlap area were analyzed by ZEN software. LDs size and number were quantified with Image J software.

Cell imaging, analysis and statistics

Cells in the Petri dish after 6-OHDA induced at the pointed time intervals (0, 30, 60, 90, 120, 150, 180, 210, 240, 270, 300 min) were co-stained with Hochest 33342 (for nucleus), 2-DPAN (lipid droplets) and Mito Tracker (mitochondria) for 20 min, and then washed with PBS for 3 times. The images were obtained using the confocal microscope (Zeiss, Germany) with emission filter at 405 nm, 405 nm and 530 nm, respectively. For oleic acid pre-treated group, the same parameters were set as above. Overlap area was analyzed by ZEN software. LDs size and number were quantified with Image J software. Data were expressed as means ± SEM. Analysis was performed using SPSS 19.0 software (IBM). One-way analysis of variance was performed. The significance level was $p<0.05$.

Mitochondrion membrane potential assay

The changes of mitochondrion membrane potential were carried out using JC-10 potential assay kit (Life, USA). Briefly, cells in the Petri dish after 6-OHDA induced at the pointed time intervals (0, 30, 60, 90, 120, 150, 180, 210, 240, 270, 300 min) were co-stained with JC-10 (5 μM) for

30 min and then captured in confocal microscope (Zeiss, Germany) with emission filter at Ex/Em= 490/525-532 nm (green); Ex/Em= 533/590-700 nm (red). To quantification the fluorescence intensities changes, cells were seeded on 96-well plate (5×10^4 cells per mL) overnight, and then 6-OHDA (100 μM) treated with the cells in different time intervals (0, 30, 60, 90, 150, 180, 210, 240, 270, 300 min). After discarding the supernatant, cells were cocultured with JC-10 for 30 min and then washed with HBSS, then 100 μL HBSS was added to each well and read in a fluorescence microplate reader (Tecan pro2000. green, Ex/Em= 490/525 nm; red, Ex/Em= 540/590 nm.)

Inflow microscopy

Cells were seeded on 6-well plate (5×10^4 cells per mL) overnight, and then 6-OHDA (100 μM) treated with the cells in different time intervals (0, 30, 60, 90, 150, 180, 210, 240, 270, 300 min). After discarding the supernatant, cells were cocultured with 2-DPAN and Mito Tracker in medium with 1% FBS for another 20 min. Cells were washed three times with PBS and collected for ImageStream analysis (Amnis, Seattle, USA). The selected channels at Ch1 (SSC channel to avoid debris and for bright field), Ch3 (561 nm excitation, 560-595 emission, for visualizing mitochondria), Ch8 (405 nm excitation, 505-560 nm emission, for visualizing LDs). To make the results more accurate, 10,000 cells in samples and 500 cells in compensation samples were acquired.

Lipase and oleic acid treatment

Lipase (10 mg/mL) and OA (1 mM) were pre-incubation with SH-SY5Y cells for 6 h, respectively. Then the cells were treated with 6-OHDA (100 μM) to induce PD model. The cell viability was evaluated by MTT assay and the LDs and cell changes were observed using confocal microscopy.

Reactive oxygen species detection

OA (0, 1 mM) were pre-incubation with SH-SY5Y cells for 6 h in Petri dishes and 96-well plate. Then the cells were treated with 6-OHDA (100 μM) for 0, 30, 150 min, and then co-cultured with 2’,7’-Dichlorofluorescin diacetate (DCFH-DA, 1 μM) for 20 min, cells were captured under confocal microscope with excitation filter at 488 nm, excitation wavelength at 522 nm. Cells in 96-well were under the similar process and reading in the microplate reader with excitation 488 nm, emission 522 nm.
